# Investigating the Synergistic Effects of Combined Modified Alginates on Macrophage Phenotype

**DOI:** 10.3390/polym8120422

**Published:** 2016-12-06

**Authors:** Hannah C. Bygd, Kaitlin M. Bratlie

**Affiliations:** 1 Department of Materials Science & Engineering, Iowa State University, Ames, IA 50011, USA; hcbygd@iastate.edu; 2 Department of Chemical & Biological Engineering, Iowa State University, Ames, IA 50011, USA; 3 Division of Materials Science & Engineering, Ames National Laboratory, Ames, IA 50011, USA

**Keywords:** alginate, macrophage phenotype, TNF-α, synergy

## Abstract

Understanding macrophage responses to biomaterials is crucial to the success of implanted medical devices, tissue engineering scaffolds, and drug delivery vehicles. Cellular responses to materials may depend synergistically on multiple surface chemistries, due to the polyvalent nature of cell–ligand interactions. Previous work in our lab found that different surface functionalities of chemically modified alginate could sway macrophage phenotype toward either the pro-inflammatory or pro-angiogenic phenotype. Using these findings, this research aims to understand the relationship between combined material surface chemistries and macrophage phenotype. Tumor necrosis factor-α (TNF-α) secretion, nitrite production, and arginase activity were measured and used to determine the ability of the materials to alter macrophage phenotype. Cooperative relationships between pairwise modifications of alginate were determined by calculating synergy values for the aforementioned molecules. Several materials appeared to improve M1 to M2 macrophage reprogramming capabilities, giving valuable insight into the complexity of surface chemistries needed for optimal incorporation and survival of implanted biomaterials.

## 1. Introduction

Macrophages are functionally diverse cells with a multitude of roles in immunity [1], disease [2], and wound healing [1,3,4,5], making them an appealing target in research areas like drug delivery [6,7], regenerative medicine [5], and biomaterial implantation [8]. In particular, they are crucial in the initiation, propagation, and resolution stages of the foreign body response (FBR) to implanted biomaterials [9,10,11]. Macrophages are important in all stages of this response due, in part, to their plasticity and heterogeneous phenotypes. The phenotypes in which macrophages exist are best described as a complex scale, bookended by classical and alternative activations.

Classically activated, M1, macrophages are said to be pro-inflammatory and cytotoxic [12,13]. As cells that mediate immune responses to bacterial, viral, and fungal infections, they can be activated by microbial stimuli (lipopolysaccharides (LPS)) or interferon (IFN)-γ, which is released by activated lymphocytes [14,15,16]. The inflammatory response that these stimulating factors initiate is followed by the release of important cytotoxic molecules such as reactive nitrogen intermediates (RNIs) and tumor necrosis factor (TNF)-α that can have an impact on the presence of tumor cells [8,13,16]. The functions of M1 macrophages also make them important in the inflammation stages of wound healing and the FBR, where they produce pro-inflammatory cytokines, phagocytose microorganisms near the injury, and recruit additional inflammatory cells to the site [8,10,17,18,19]. Alternatively activated, M2, macrophages are known to be pro-angiogenic and can be activated by interleukin (IL)-4 or IL-13 [20]. The main functions of these cells include: maintaining homeostasis, tissue repair and remodeling, as well as promoting wound healing in the resolution stage of the FBR [8]. They fulfill these roles with the release of IL-10, vascular endothelial growth factor (VEGF), and transforming growth factor (TGF)-β [21]. The entire spectrum of phenotypes that exists is imperative in one way or another [4,8], but an increased presence of either M1 or M2 macrophages may be useful for specific applications. For example, an earlier M2 macrophage presence rather than prolonged M1 presence may allow for proper wound closure, but in cancer therapies an M1 macrophage presence rather than pro-angiogenic, tumor associated M2 like macrophage, may lead to the elimination of cancerous cells.

With the application of wound healing and successful implant incorporation, the significance of macrophage plasticity is made evident by examining the timeline of macrophage presence during the FBR [8]. Macrophage phenotype is dynamic throughout the entirety of the FBR, and a balance of phenotypes is essential for timely progression from injury to proper healing [8]. After implantation of a biomaterial, macrophages are classically activated by proteins that have adsorbed to the material surface [22,23,24]. These macrophages release pro-inflammatory cytokines that continue to attract monocytes to the injury site leading to chronic inflammation [19]. Attempting and failing phagocytosis of the large implant, causes the M1 macrophages to fuse into foreign body giant cells (FBGC) [25]. Chronic inflammation is only resolved with a presence of alternatively activated macrophages resulting from phagocytosis of dying cells around the implant, or stimulation by IL-4 or IL-13, typically from basophils [20,26,27]. M2 macrophages seek to promote wound healing in the area of the implant [11]. However, prolonged M2 macrophage activity can lead to the formation of an extensive fibrous capsule that may negatively impact the function of the implant [28]. Finding a balance between chronic inflammation and continued wound healing remains an issue with many biomedical implants. Using the idea that material surface chemistries have an impact on macrophage phenotype, it may be hypothesized that a particular material could orchestrate appropriate progression through the FBR to avoid both chronic wound healing and complete fibrous encapsulation. This may be particularly applicable for electrospinning coaxial fibers [29] and sensors [30], in addition to scaffolds for tissue engineering applications [31].

Many techniques for macrophage reprogramming have been developed that focus on either the chemical or environmental stimuli to which macrophages are exposed [14,15,32,33]. Previous work in this lab has indicated that some chemically modified alginates may impact macrophage phenotype [9]. For example, some modifications decreased TNF-α production by M1 macrophages, which could make them useful for decreasing the inflammatory response to a material. Other modifications appeared to amplify the presence of M2 macrophages by showing an increased arginase/iNOS (inducible nitric oxide synthase) ratio compared to the control. Some of the most promising materials showed decreased TNF-α production and increased arginase/iNOS ratios for all activations of macrophages. Finally, some indicated that even a small amount of modification may be giving the materials some permselectivity capabilities, which could be important for applications in drug delivery and tissue engineering.

Given the complexity of biological systems and the immune system in particular, the idea that the most effective material surface might be made up of several chemistries is reasonable [34]. However, this can make optimal biomaterial design criteria difficult to ascertain [34]. Cellular responses to materials are typically directed by surface protein adsorption [22], and many biological interactions, such as protein–ligand or protein–cell interactions, are polyvalent in nature [22,35]. This suggests a need for a material with multiple surface chemistries to more closely imitate natural interactions, as multiple simultaneous interactions may have unique collective properties that vary from those displayed by each component individually [35]. The synergistic effects of material properties have typically been studied using high-throughput combinatorial approaches [36]. This logic and testing method have similarly been employed in synergistic drug delivery, where the aim is to achieve some synergistic therapeutic effect, reduction of dose or toxicity, and to delay or minimize drug resistance [37,38,39,40].

The focus of this work was to examine the synergistic effects of a few promising material surface chemistries described above. In order to more accurately describe the phenotype of macrophages in vitro, a newer nomenclature will be used throughout this paper that is based on the molecule used to activate the cells, for example M(LPS) and M(IL-4) [12]. By modifying alginate with one functional group that amplified M(IL-4) activation (ester or oxime) and one that indicated M(LPS) to M(IL-4) macrophage reprogramming (amide or nitro), the goal was to achieve an even greater M(IL-4) response from all macrophage phenotypes [9]. This could be useful in the proper integration of various biomaterials. Other materials examined here combine a surface modifier that had high permeability but low production of toxic cytokines (sulfonic acid or nitro) with one that had low permeability but high cytokine production (ketal or epoxide) [9]. An ideal combination of modifications would have low permeability and also cause low production of toxic cytokines. Understanding the synergistic effects of these materials may lead to the development of a diverse library of materials that could influence any desired macrophage phenotype.

## 2. Materials and Methods

Experiments were performed with a minimum of three replicates. Results were compared to controls of unmodified alginate as well as singly modified samples in previous work [9]. All materials were purchased from Sigma (St. Louis, MO, USA) and used as received, unless otherwise indicated. Fresh deionized (DI) water (Milli-Q Nanopure, Thermo Scientific, Waltham, MA, USA) was used throughout this study.

### 2.1. Materials

Surface modifiers [9], coupled to medium viscosity (~600–900 cps, ~250,000 *M*_W_) alginate (MP Biomedicals, Santa Ana, CA, USA) were chosen based on the results of previous research in this lab [9]. This included: glycidamide; *tert*-butyl 4 aminobutanoate (VWR, Radnor, PA, USA); malonamide (Fisher, Pittsburgh, PA, USA); 1-amino-4-oxocyclohexane carboxylic acid ethylene ketal; 2,4-dinitro-phenyl-hydoxylamine; 3-aminobenzamide oxime; and 3-amino-1-propane sulfonic acid (Fisher, Pittsburgh, PA, USA).

### 2.2. Alginate Modification

The seven different surface modifiers were combined and coupled to the medium viscosity alginate using 1-ethyl-3-(3-dimethylaminopropyl)carbodiimide (EDC, Oakwood Chemical, West Colombia, SC, USA) and *N*-hydroxysuccinimide (NHS, Thermo Scientific, Waltham, MA, USA). These materials were modified as previously described [9] with 50 molar equivalents of modifier 1 and 50 molar equivalents of modifier 2, rather than 100 molar equivalents of one modifier.

### 2.3. Elemental Analysis

In order to determine the percent modification of each material, elemental analysis was performed to obtain %C, %H and %N. Measurements were recorded in triplicate with an acetanilide calibration standard, combustion and reduction temperatures of 925 and 640 °C, respectively, and a resulting accuracy of ±0.3% for each element. All standards and reagents are from Perkin Elmer and/or Elementar America’s Inc. (Mt. Laurel, NJ, USA). The instrument used was a PE 2100 Series II combustion analyzer (Perkin Elmer Inc., Waltham, MA, USA).

### 2.4. Water Contact Angle (WCA)

Samples were prepared by creating a positively charged surface on glass microscope slides with poly-l-lysine (PLL). This allowed for a thin, even coating of each modified alginate to be applied to slides by crosslinking with SrCl_2_ (Alfa Aesar, Haverhill, MA, USA). These coated slides could then be inverted over a container of water. An air bubble (100 μL) was deposited under the slide and imaged using a digital camera (Canon EOS Rebel T3i, Canon, Melville, NY, USA). Five replicates were collected for each sample before the angle between the slide and the bubble was measured using ImageJ (NIH, Bethesda, MD, USA) software. The final reported WCA is 180° minus the measured angle.

### 2.5. Compression Modulus

The compression modulus was measured for each sample using manual compression testing techniques. Modified alginate pegs were made to be approximately 10 × 10 × 4 mm^3^ in size. These pegs were placed between two microscope slides and imaged after each of various sized weights were set on the top slide. The changing distance between the slides in each image was measured using ImageJ, and used to generate stress-strain curves. Linear portions of these curves were used to determine the reported compression modulus for each sample (*n* = 5).

### 2.6. Cell Culture

RAW 264.7 cells (American Type Cell Collection, ATCC, Manassas, VA, USA) were used as a model cell line for macrophages in these experiments. Cells were cultured in complete medium (CM, Dulbecco’s modified Eagle’s medium (DMEM, Mediatech, Inc., Manassas, VA, USA)) supplemented with 10% fetal bovine serum (FBS, Mediatech, Inc., Manassas, VA, USA), 100 U/mL penicillin, and 100 μg/mL streptomycin) at 37 °C in 5% CO_2_. Every three to five days, the cells were passaged using a cell scraper to detach cells and subcultured between ~6.7 × 10^3^ and 2.7 × 10^4^ cells/cm^2^.

### 2.7. Cell Viability

In order to test the viability of RAW 264.7 cells in contact with the combined modified alginates, plates were seeded for MTT (methyl thiazolyldiphenyl tetrazolium, Research Products International Corp., Mt Prospect, IL, USA) assays during passaging. For this, 24-well plates (KSE Scientific, Durham, NC, USA) were first coated with 0.05% PLL solutions using 200 μL/well and then incubated at 37 °C for 1 h. Before seeding the cells into the plate, each well was washed twice with sterile phosphate buffered saline (PBS). The plates were seeded with 125,000 cells/cm^2^ in 500 μL of CM per well and 25 ng/mL IL-4 (M(IL-4)) (eBioscience Inc., San Diego, CA, USA) or 5 ug/mL LPS (M(LPS)) and the cells were allowed to adhere for 24 h. A control set of experiments using non-activated cells was included and referred to as naïve or M(0) cells. Alginate coatings were created by adding ~100 μL of modified alginate to each well, allowing it to coat the bottom, and excess alginate was removed. Next, 500 μL of 0.2 M SrCl_2_ were used per well to crosslink the coating. This solution was left in the wells for approximately 5 min before replacing it with clear CM. After 48 h the supernatant was removed and saved for further testing. To each well 500 μL of clear CM was added along with 50 μL of MTT (5 mg/mL in DI water). After a 2 h incubation at 37 °C, 425 μL of the solution was removed from each well and replaced with 500 μL of dimethyl sulfoxide (DMSO). The absorbance of each plate was read at 540 nm with a reference of 690 nm using a BioTek Synergy HT Multidetection Microplate Reader (BioTek, Winooski, VT, USA). Positive controls for each plate were cells with no alginate samples, and negative controls contained media, modified alginates, and activators. Experiments were performed in quadruplicate and results are given as the mean value for each sample normalized to the positive control ± standard deviation.

### 2.8. TNF-α ELISA

Measurement of TNF-α, an M1 phenotype marker, was performed using commercially available enzyme linked immunosorbent assay (ELISA) kits (eBioscience, Inc., San Diego, CA, USA) and performed as described by the manufacturer, using the supernatant collected during viability assays.

### 2.9. Urea Assay

After 48 h of incubation with the combined modified alginates, the supernatant was removed and saved for further testing. Cells were then washed with 400 μL of PBS, and the plates were placed on ice for 10 min with 100 μL of cell lysis buffer (150 μL protease inhibitor cocktail (Amresco, Solon, OH, USA) and 15 μL Triton X-100 (Acros Technologies, Elgin, IL, USA) diluted to 15 mL with DI water) per well. The resulting lysate (25 μL) was transferred from each well to a 96-well plate (Argos Technologies, Elgin, IL, USA) along with 25 μL of a 10 mM MnCl_2_ (Fisher, Pittsburgh, PA, USA) and 50 mM Tris solution (Fisher, Pittsburgh, PA, USA). The plate was incubated for 10 min at 55 °C before adding 50 μL of 1 M arginine (pH 9.7) to each well and incubating at 37 °C for 20 h. Arginase activity, an M2 phenotype indicator, was measured through the conversion of arginine to urea. This was done by adding 200 μL of a 1:2 ratio of solution 1 (1.2 g *o*-phthaldialdehyde (Alfa Aesar, Haverhill, MA, USA), 1 L H_2_O, and 500 μL HCl (Fisher, Pittsburgh, PA, USA)) and solution 2 (0.6 g *N*-(naphthyl)ethylenediamine dihydrochloride (Acros Technologies, Elgin, IL, USA), 5 g boric acid (Fisher, Pittsburgh, PA, USA), 800 mL H_2_O, 111 mL sulfuric acid (Fisher, Pittsburgh, PA, USA), diluted to 1 L with H_2_O) [41]. The plate was read at 520 nm with a reference at 630 nm.

### 2.10. Griess Reagent Assay

Nitrite production, which is also indicative of an M1 phenotype, was measured from the supernatant collected in the urea assay described above. A standard curve was created using serial dilutions of 100 μM NaNO_2_ with volumes of 150 μL. To the remaining wells 150 μL of sample were added. To the entire plate, 130 μL of DI water and 20 μL of Griess reagent (Acros Technologies, Elgin, IL, USA) were added and allowed to incubated for 20 min. The plate was read at 448 nm with a 690 nm reference.

### 2.11. Immunocytochemistry

RAW 264.7 cells were fluorescently labeled using a previously developed protocol [9]. Briefly, cells were seeded at 100,000 cells/cm^2^ on clean, PLL coated glass coverslips in Petri dishes, as M(LPS), M(IL4), or M(0) and were coated with pairwise modified alginates as described above. After 48 h of incubation, the cells were stained for CD11c and CD206. These coverslips were imaged with an EVOS^®^ FLoid^®^ Imaging Station (Life Technologies, Grand Island, NY, USA) using the red channel (excitation/emission 586/646 nm), blue channel (390/446 nm) and the green channel (482/532 nm).

### 2.12. TNF-α Diffusion

To test the diffusive properties of these pairwise modified alginates, microparticles were made using an electrostatic droplet generation technique. Using the same equipment and techniques previously described [9], microparticles with an average diameter of 685.15 ± 14.20 μm were made with unmodified alginate. Amino polystyrene particles, 10 mg, (Spherotech Inc., Lake Forest, IL, USA) were also labeled with 2 mg mouse IgG (Thermo Scientific, Waltham, MA, USA) in PBS using 20 mg of EDC. These labeled polystyrene particles were encapsulated in the unmodified alginate particles during electrostatic droplet generation using procedures developed by Kulseng et al. [42]. Approximately 200 particles were incubated with 2 mL of supernatant known to contain TNF-α. This supernatant was collected from M(LPS) RAW 264.7 cells cultured at ~182,000 cells/cm^2^ after two days of incubation. After four days of incubation with the particles, the supernatant was removed and compared to the original TNF-α supernatant using the TNF-α ELISA described in Section 2.8 to determine how much had diffused into the particles.

### 2.13. Statistics and Data Analysis

Statistical analysis was performed using JMP^®^ statistical software (Cary, NC, USA). Statistical significance of the mean comparisons was determined by a two-way ANOVA. Pair-wise comparisons were analyzed with Tukey’s honest significant difference test. Differences were considered statistically significant for *p* < 0.05.

## 3. Results

### 3.1. Modification and Characterization

**Figure 1:**
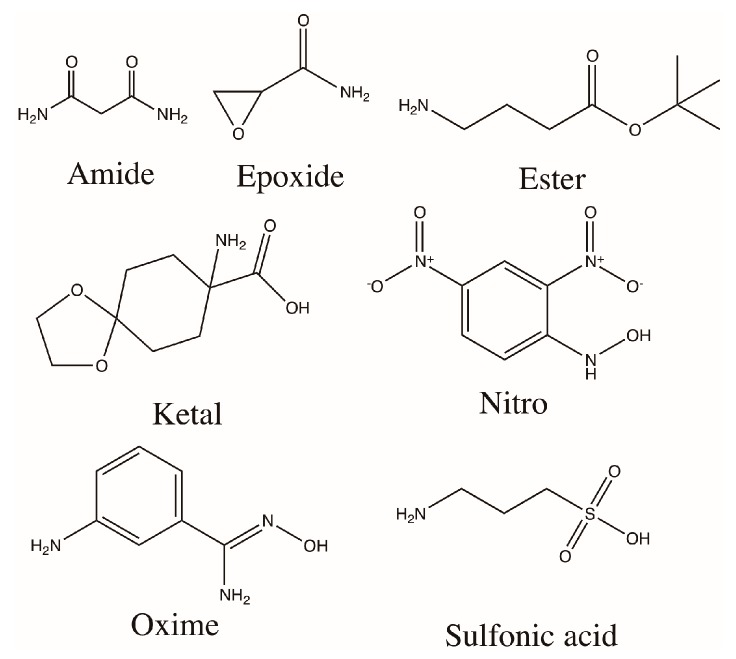
Chemical structures of the molecules used in the modification of medium viscosity alginate. The functional groups listed here are used as labels in the following figures for convenience.

**Figure 2:**
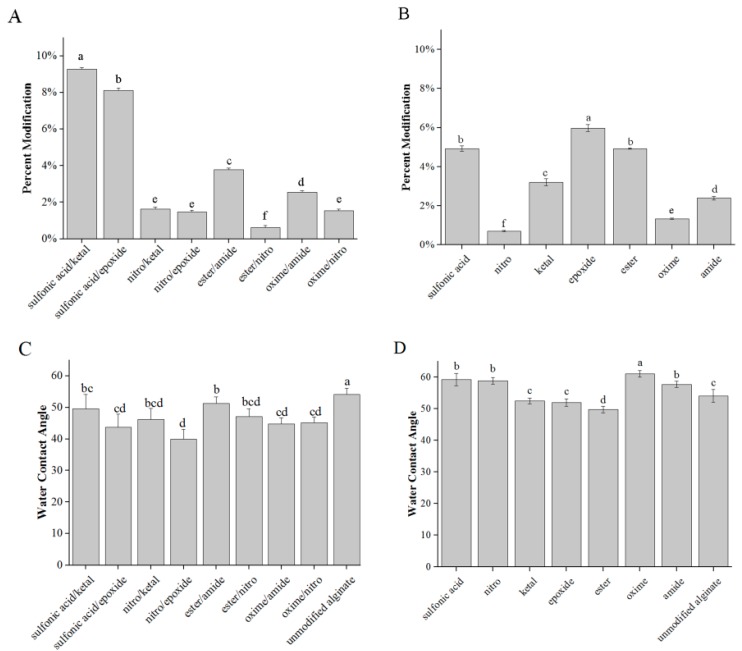

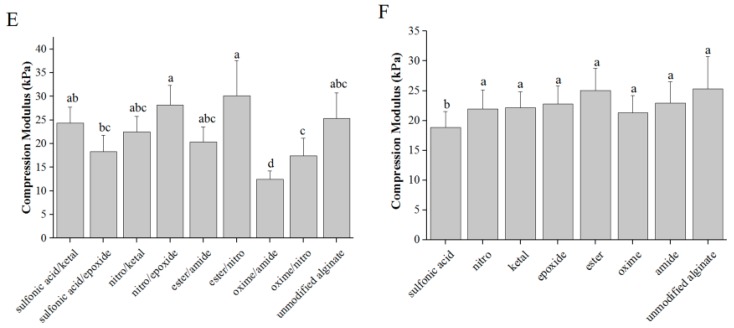
Characterization of combined and single modified alginates: (**A**,**B**) percent modifications; (**C**,**D**) WCAs; and (**E**,**F**) compression moduli were measured for double (**A**,**C**,**E**) and single (**B**,**D**,**F**) modifications. *n* = 5. Data represent the mean value ± standard deviation. Bars with the same letter (a–f) are not statistically different (*p* < 0.05).

After the combined modification of alginate using the molecules in Figure 1, the synthesized materials were characterized for their hydrophobicity, compression modulus and percent modification. Elemental analysis confirmed modification with %N values being greater for the modified alginates than unmodified (Figure 2A,B). The WCAs of the combined modified alginates fell in a range of 39.9°–51.2° (Figure 2C), compared to the range of 49.7°–61.0° with the single modifications, and 54.1° ± 2.0° for unmodified alginate(Figure 2D). This suggests the materials remained relatively hydrophilic. The compression moduli of the combined modified alginates ranged from 12.5–30.1 kPa (Figure 2E), which was similar to the range in compression modulus for the single modifications (18.8–25.0 kPa), and of unmodified alginate (25.3 ± 5.4 kPa, Figure 2F).

### 3.2. Cell Viability

**Figure 3:**
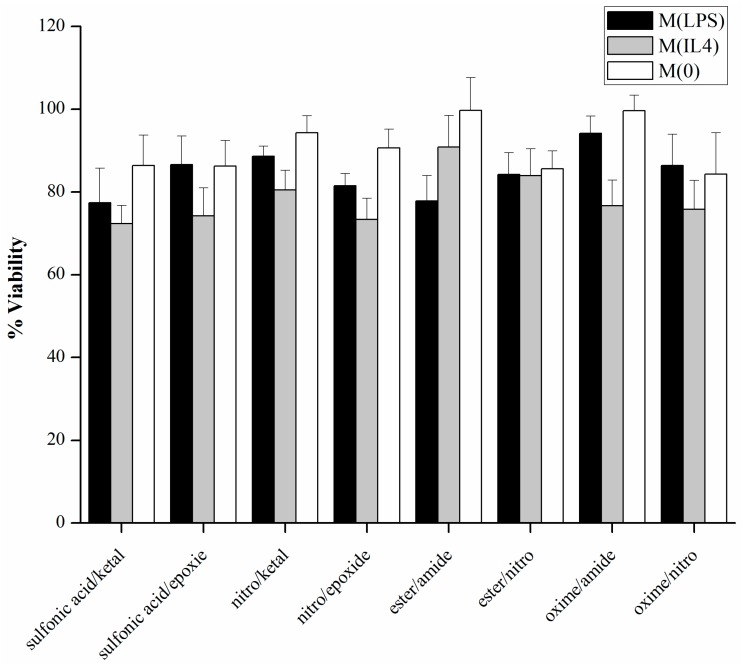
Modified alginates are cytocompaible. Cell viability of M(LPS), M(IL-4) and M(0) RAW 264.7 macrophages encapsulated under modified and unmodified alginate layers. *n* = 4. Data represent the mean value ± standard deviation.

Biomaterials must be cytocompatible for use as implantable materials. Cytotoxicity of the combined modified alginates was measured using MTT assays. The viability of the cells was expressed as a percentage of the positive control of cells cultured on PLL coated tissue culture plastic. Cells exposed to all materials showed greater than 70% viability (Figure 3). This suggests minimal cytotoxicity, especially for cells cultured under a hydrogel.

### 3.3. Arginase/iNOS

**Figure 4:**
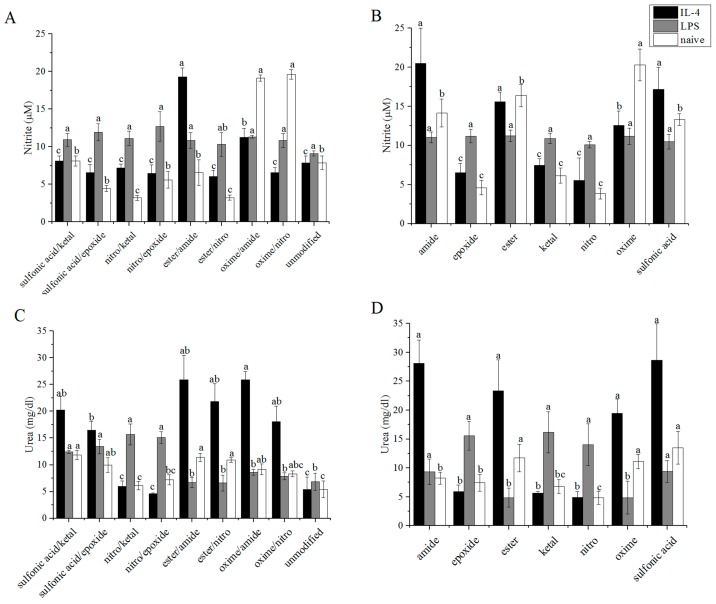

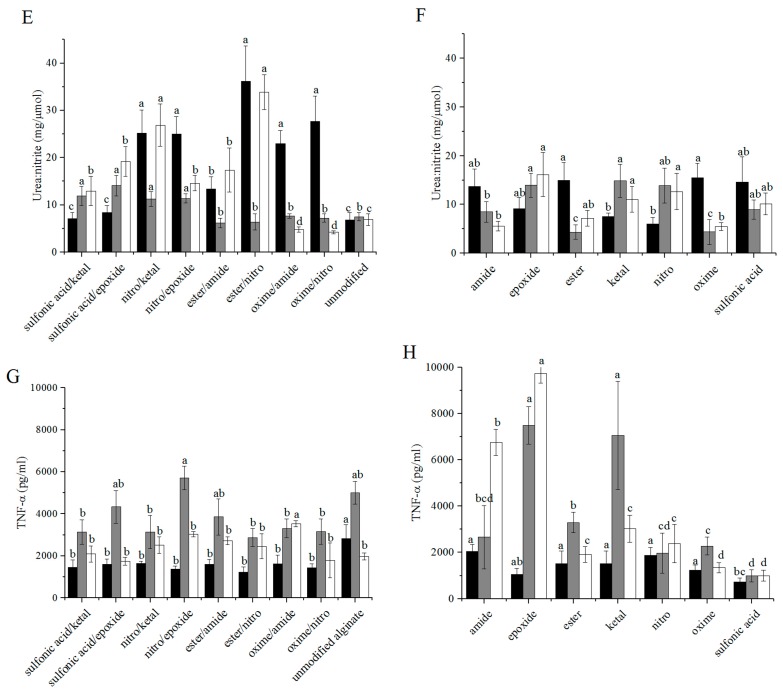
Molecules produced by polarized RAW 264.7 macrophages encapsulated under modified alginates. Alginates were layered over activated macrophages and: (**A**,**B**) nitrite; (**C**,**D**) urea; (**E**,**F**) urea:nitrite; and (**G**,**H**) TNF-α were measured for dual (**A**,**C**,**E**,**G**) and single (**B**,**D**,**F**,**H**) modifications. Arginase activity was measured by removing the alginate layer and lysing the cells. *n* = 4. Data represent the mean value ± standard deviation. Bars with the same letter (a–d) are not statistically different (*p* < 0.05) from data points of the same activation.

Arginase activity was determined for encapsulated macrophages by exposing cell lysate to arginine and quantifying the urea produced. Nitrites are the stable form of NO and were quantified through a Griess reagent assay. Absolute values for both urea and Griess assays are given in Figure 4A–D, with the pairwise modifications on the left and the single modifications on the right for comparison. Results from these assays are given as urea: nitrite in Figure 4E,F, which corresponds to the amount of urea produced divided by the amount of nitrite in corresponding samples. Ratio values range from 7.12 mg/μmol with sulfonic acid/ketal to 36.2 mg/μmol with ester/nitro for M(IL-4) macrophages. A value of 6.88 ± 3.05 mg/μmol was previously determined for M(IL-4) macrophages in the presence of unmodified alginate [9]. M(LPS) macrophages exhibited a narrow range of 6.20 to 14.1 mg/μmol compared to 7.52 ± 1.76 mg/μmol for unmodified alginate. Finally, values for M(0) macrophages ranged from 4.24 mg/μmol with the oxime/nitro modification to 33.8 mg/μmol with ester/nitro. M(0) macrophages exhibited a urea:nitrite ratio of 6.91 ± 2.13 mg/μmol with unmodified alginate. Higher values for these ratios indicate a stronger M2 phenotype presence, while low values suggest a M1 phenotype. Most values seem to indicate a stronger M2 presence when compared to unmodified alginate. Exceptions to this statement include oxime/amide and oxime/nitro M(LPS) macrophages.

The controls for M(IL-4), M(LPS), and M(0) cells were higher than the cells exposed to the modified and unmodified alginates with 124 ± 30.6, 13.1 ± 2.38 and 94.3 ± 29.5 mg/μmol being measured, respectively. The urea measured for the controls was in line with the values measured for the cells exposed to alginate samples—20.1 ± 3.25, 16.4 ± 2.97 and 6.31 ± 1.14 mg/dL for M(IL-4), M(LPS), and M(0) cells, respectively. However, the nitrite levels were much lower for M(IL-4) and M(0) cells, leading to a large increase in the urea:nitrite ratio. The measured nitrite levels were 1.62 ± 0.30, 12.5 ± 0.25 and 0.67 ± 0.17 μM for M(IL-4), M(LPS), and M(0) cells respectively.

### 3.4. TNF-α ELISA

Production of the cytotoxic cytokine TNF-α by cells exposed to modified alginates was measured and reported in Figure 4G. Values for single modifications of alginate are included in Figure 4H for comparison. Most modifications of alginate were able to decrease the amount of TNF-α produced in comparison to unmodified alginate for all macrophage phenotypes. The exception to this statement is oxime/amide for M(0) cells. TNF-α production by M(IL-4) macrophages ranged from 1.2 to 1.6 ng/mL with 2.8 ± 0.68 ng/mL produced under unmodified alginate. M(0) macrophages produced a range of TNF-α that spanned 1.8 to 3.5 ng/mL with unmodified alginate eliciting 1.9 ± 0.17 ng/mL TNF-α. TNF-α produced by M(LPS) cells spanned a higher range of 2.9 to 5.7 ng/mL, and on a sample-to-sample basis, the amount of cytokine produced was most often highest for M(LPS) macrophages. In the presence of unmodified alginate, M(LPS) cells produced 5.0 ± 0.53 ng/mL of TNF-α. All modifications had statistically lower TNF-α production than unmodified alginate for M(IL-4) macrophages. Similar to nitrite, TNF-α secretion from M(IL-4) and M(0) cells was much lower for the control than for the cells in the presence of alginate. The values for the controls were 0.58 ± 0.066, 5.0 ± 0.35 and 0.39 ± 0.016 ng/mL for M(IL-4), M(LPS) and M(0) cells, respectively.

### 3.5. Fluorescent Imaging

**Figure 5:**
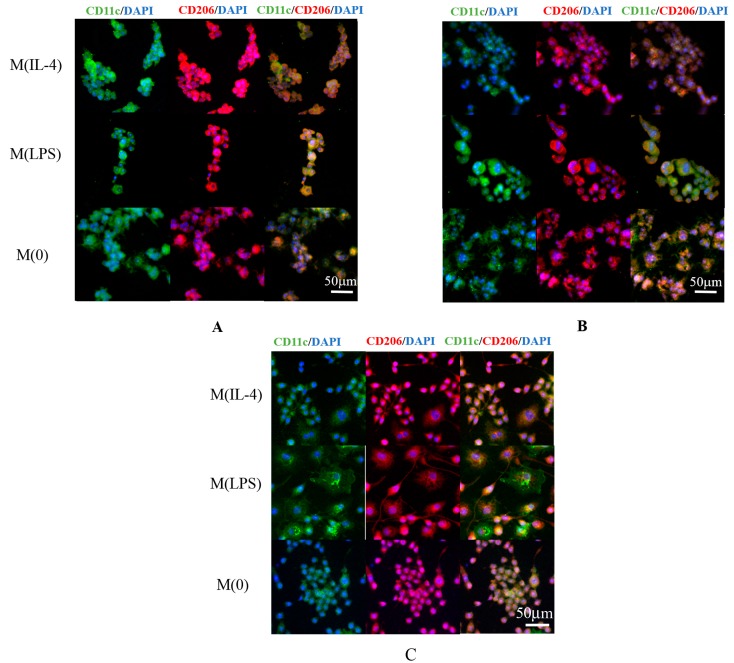
Immunocytochemistry staining of macrophages suggests that modified alginates alter phenotype: (**A**) nitro/epoxide; and (**B**) ester/amide modified alginates were coated on cells; and (**C**) cells in the absence of alginate are shown for comparison. Cells were stained with fluorescently labeled CD206 (red, M2 marker) and CD11c (green, M1 marker) markers as well as DAPI (blue). Scale bar is 50 μm.

Immunocytochemistry (ICC) fluorescent staining was used to identify the macrophage phenotypes resulting from culturing cells in the presence of modified alginates. CD11c, an LPS receptor, was used to identify M1 macrophages and a CD206, a mannose receptor, was used to indicate an M2 macrophage phenotype. DAPI was also used as a nuclei stain. Representative images are shown in Figure 5A,B. These images were used to qualitatively support the results found by measuring arginase activity, TNF-α secretion, and nitrite production. Control staining images of macrophages with activator alone are also included in Figure 5C.

### 3.6. Synergy

**Figure 6:**
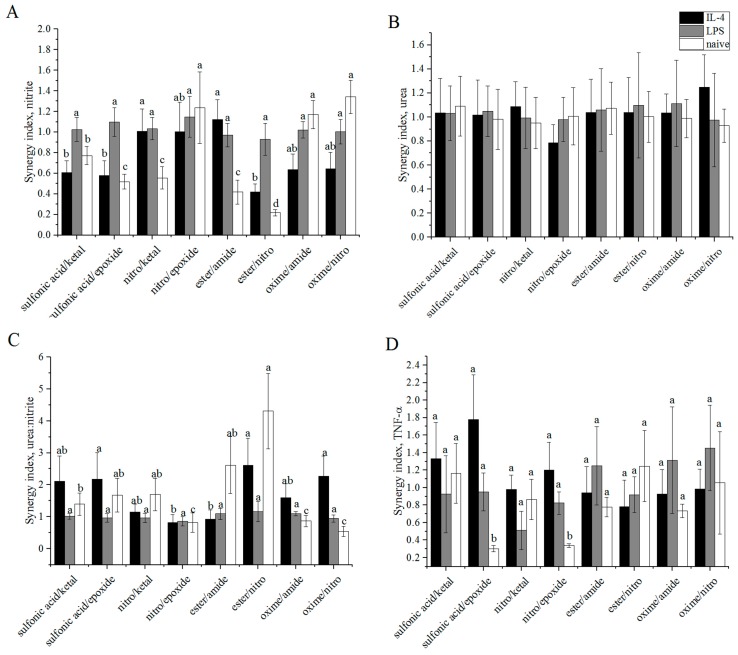
Synergistic indices for molecules produced by polarized RAW 264.7 macrophages: (**A**) nitrite; (**B**) urea; (**C**) urea:nitrite; and (**D**) TNF-α for M(LPS), M(IL-4), and M(0) cells were calculated based on previously obtained values for single modifications of alginates [9]. *n* = 4. Data represent the mean value ± standard deviation. Bars with the same letter (a–d) are not statistically different (*p* < 0.05) from data points of the same activation. All measured values in panel B were statistically similar.

Normalizing the percent modifications of each combined alginate sample to that of the single modifications, we were able to determine weight fractions of each modification. Synergy was calculated using the equation that was developed by Karande et al. [43]:

(1) $$S=\frac{m_{A+B}}{xm_{A}+(1-x)m_{B}}$$

where *m*_A_, *m*_B_, and *m*_A*+*B_ are the measured values for the different modifications and *x* is the weight fraction of each modification determined by elemental analysis. These values are shown for each macrophage phenotype in Figure 6A–D. The synergy values for nitrite were nearly one for M(LPS) cells exposed to all modifications (0.930–1.147). Synergy values were slightly more dynamic for M(IL-4) cells, ranging from 0.578 to 1.121, and for M(0) cells, ranging from 0.218 to 1.34. For urea, all of the synergy values for all of the macrophage activation states were near unity. This resulted in nitrite being entirely responsible for the synergy values for urea: nitrite. Since M(LPS) cells did not have large variation in the synergy values for the amount of nitrite measured in the supernatant, the synergy values were correspondingly nearly unity (0.854–1.159). Both M(IL-4) and M(0) cells yielded more dynamic synergy values for urea: nitrite of 0.811 to 2.613 for M(IL-4) and 0.533 to 4.31 for M(0). Finally, the synergy values for TNF-α production were in the range of 0.783–1.778 for M(IL-4) macrophages, 0.511–1.453 for M(LPS) macrophages, and 0.303–1.246 for M(0) macrophages.

### 3.7. TNF-α Diffusion

**Figure 7:**
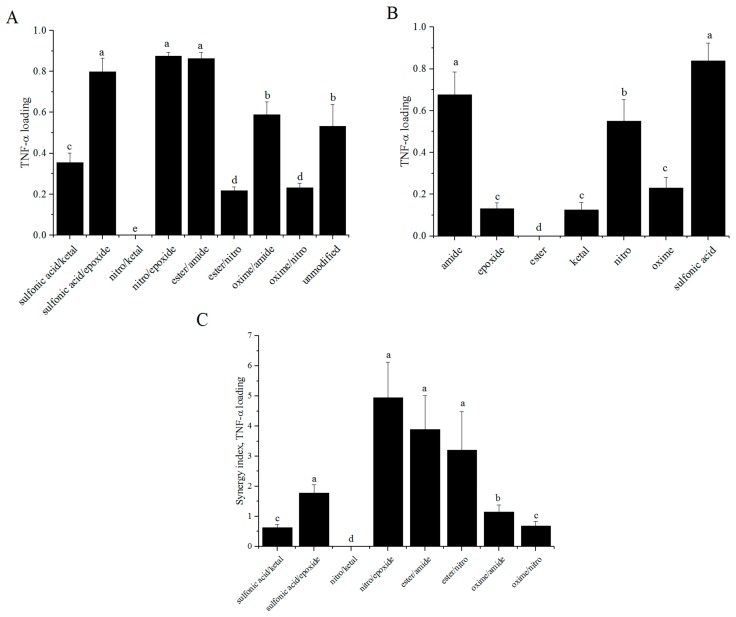
Modified alginates alter mass transport of TNF-α through layered alginate particles. (**A**) Combined; and (**B**) single modified alginates were layered over alginate particles containing mouse IgG labeled polystyrene particles. *n* = 4. Data represent the mean value ± standard deviation. Bars with the same letter (a–d) are not statistically different (*p* < 0.05) from data points of the same activation.

The amount of TNF-α able to diffuse into the particles was measured using an ELISA after four days of incubation (Figure 7A) [9]. A wide range of values were calculated for percent loading. Nitro/ketal did not allow any diffusion of TNF-α, while nitro/epoxide allowed 87% loading. Percent loading values for single modifications of alginate are included in Figure 7B for comparison. Synergy values for TNF-α diffusion were calculated in the range of 0 to 4.94 (Figure 7C).

## 4. Discussion

Alginate is an appealing biomaterial for numerous wound healing and tissue engineering applications. It is a naturally occurring, relatively inexpensive polymer, it has low toxicity and good biocompatibility, and is easily gelled under mild conditions with divalent cations [44]. The structure of alginate is similar to that of the extracellular matrix in living tissues, and it provides a relatively inert and moist microenvironment [44,45] However, the biocompatibility of alginate remains insufficiently understood. For example, in research by Elliot et al., alginate was used to encapsulate porcine islets in a clinical trial for diabetes treatment [46]. While the trials were mildly successful in that there were surviving functional islets, the overall function of the implant was severely diminished by the presence of granulation tissue that developed in response to the alginate [46]. Many aspects of the polymer have been studied in an attempt to improve upon the biocompatibility [47,48,49,50,51,52,53,54,55,56,57,58,59,60], and some work has been done to chemically modify alginate and study the impact this has on cell function [9,61,62,63]. This research focuses on combining surface modifications to study the synergistic effects of chemical modification on cellular responses. By using materials that have been studied in the past, we have been able to compare predicted and actual values to examine synergy of the modifications.

In this study, we aimed to find combined modifications that would improve the biocompatibility of alginate by reprogramming macrophages towards an M2-like phenotype. Based on the equation used to calculate synergy, values greater than one for urea:nitrite and less than one for both TNF-α and nitrite production would be desired. These values are most important for M(LPS) macrophages as this is the phenotype we are aiming to reprogram. M(LPS) macrophages tend to mediate the inflammatory stages of the foreign body response to biomaterials, which we are attempting to minimize. TNF-α production synergy values tended to be less than or equal to one for all phenotypes studied. This may be due, in part, to the fact the TNF-α, must diffuse through the alginate coating in order to be measured. As Figure 7 indicates, some of these combined modifications allow little to no TNF-α diffusion through the alginate. However, combining this information with the urea synergy values, we can determine which modifications may be favorably shifting macrophage phenotype towards an M2-like state. From the values presented in Figure 6, it can be determined that almost all the modifications do not result in cooperative interactions with M(LPS) macrophages. This is particularly interesting and somewhat surprising for TNF-α production since variable diffusion of TNF-α through the different modifications was observed (Figure 7).

Several different observations were noticed when examining synergistic values of TNF-α production in concert with urea:nitrite. With respect to the M(0) macrophage synergy values, sulfonic acid/epoxide and nitro/epoxide resulted in antagonistic synergy values for TNF-α production. Perhaps unsurprisingly, the pairwise modifications with epoxide resulted in increases in TNF-α production synergy values for M(IL-4) cells. Epoxide was chosen for this study because it resulted in low permeability and high TNF-α production. Sulfonic acid combinations are also able to increase TNF-α production synergy values for M(IL-4) cells and increased urea:nitrite values. Sulfonic acid/epoxide was also able to improve urea: nitrite values, indicating the combinations of these modifications shifted M(0) cells towards an M2 phenotype. Ester combinations resulted in improved urea:nitrite values and did not have an observable synergistic effect on TNF-α for M(0) cells. Future work in examining the synergy of these materials in a dose dependent context may elucidate the relationship of these modifications on shifting macrophage phenotypes.

By including the TNF-α diffusion study, this work also examined the changes in diffusive properties of alginate due to combined surface modifications. Ideally, combined modifications would result in a lower mass transport of TNF-α than in the case of single modifications. This would be indicated by synergy values less than one. Modifications sulfonic acid/ketal, nitro/ketal, and oxime/nitro all had values below one. Ketal lowering diffusion was expected since it was previously observed to have low permeability for TNF-α [9]. Interestingly, when ketal was combined with nitro or sulfonic acid, the synergy values were less than one even though nitro and sulfonic acid were selected for their poor permselectivity towards TNF-α and low production of TNF-α. The other modifications had relatively high synergy values, suggesting that they are not permselective towards TNF-α, which could potentially be harmful for islet encapsulation as in Elliot et al. [46]. In that example, tissue development surrounding the implant cut off blood supply to the encapsulated islet preventing them from functioning properly. In this case, with increased TNF-α release, the toxic cytokine would be able to reach the islets and impact their ability to regulate blood sugar.

## 5. Conclusions

Pair-wise modifications of alginate resulted in synergistic and antagonistic responses for M(IL-4), M(LPS) and M(0) cells for both nitrite and TNF-α secretion. There was no observed advantage or disadvantage of these modifications in terms of arginase activity. In combining the data measuring the shifts in macrophage phenotype with that examining TNF-α diffusion through the modified alginates, improved materials for artificial organs can be obtained. Interestingly, certain modifications appear to influence the synergistic index for TNF-α diffusion, such as ketal, which lowered the index for both pair-wise combinations in which it was included. This observation held for material mediated shifts in macrophage phenotype, e.g., ester for TNF-α secretion from M(0) cells. These results further demonstrate the importance of chemical moieties on biomaterials in achieving appropriate cellular responses. Although this study is not conclusive, it provides valuable insight into the impact of multiple surface modifications on macrophage phenotype. This library is also not large enough to offer much in the way of justified reasoning for the synergistic effects of the combined modifications. However, it provided evidence that maximum macrophage reprogramming may require a multitude of stimuli, and further supported the findings that simply altering the surface chemistry of a biomaterial may have a significant impact on its biocompatibility.
